# Sirolimus Monotherapy for Thrombocytopenia in Primary Antiphospholipid Syndrome: A Pilot Study From a Tertiary Referral Center

**DOI:** 10.3389/fimmu.2022.857424

**Published:** 2022-03-25

**Authors:** Wenhui Xie, Lanlan Ji, Zhuoli Zhang

**Affiliations:** Department of Rheumatology and Clinical Immunology, Peking University First Hospital, Beijing, China

**Keywords:** antiphospholipid syndrome, sirolimus, thrombocytopenia, real-world evidence, response

## Abstract

**Background:**

Thrombocytopenia (TP) is considered as a warning sign of high-risk antiphospholipid syndrome (APS) and sometimes a paradoxical sign of anti-thrombosis treatment. Currently, there is an extreme paucity of effective and safe drugs for long-term management of TP in primary APS patients; therefore, we explored the efficacy and safety of sirolimus monotherapy.

**Methods:**

In this real-world study, we included 7 consecutive patients with primary APS who received sirolimus monotherapy for TP. Oral sirolimus was initiated at a dose of 1–2 mg once daily and then adjusted primarily based on clinical efficacy and tolerance, with consideration of the sirolimus trough concentration of ≤15 ng/ml.

**Results:**

Of included patients, the median age was 58 years with a median disease course of 1.5 years and 4 patients were treatment-naïve. All patients completed 6 months of sirolimus therapy with a median follow-up of 6 months (range: 6–15). All patients received sirolimus monotherapy for TP during the entire follow-up, without any additional agents. Overall, the platelet count exhibited a substantially increasing trend after sirolimus administration during the first 6 months (p < 0.001) and stability later. Specifically, the median platelet count was significantly increased from 59 × 10^9^/l before sirolimus to 90 × 10^9^/l at month 1 (p = 0.028), 131 × 10^9^/l at 3 months (p = 0.028), and 178 × 10^9^/l at 6 months (p = 0.018). Overall and complete responses were respectively achieved in 6 (85.7%) and 5 (71.4%) patients at month 6. Importantly, overall response was achieved in all 4 treatment-naïve patients. Additionally, there were different extents of decline in the titers of antiphospholipid antibodies after sirolimus treatment. Regarding safety, only one patient experienced an elevated cholesterol level with recovery after atorvastatin treatment.

**Conclusion:**

Sirolimus monotherapy confers good efficacy and tolerance for TP in primary APS patients and therefore may be considered as a first-line therapy.

## Introduction

Antiphospholipid syndrome (APS) is a systemic autoimmune disease characterized by the occurrence of vascular thrombosis or obstetrical complications in combination with the persistent presence of circulating antiphospholipid (aPL) antibodies. Thrombocytopenia (TP), one of non-criterion features of APS ranging from 15% to 53%, is currently considered to be of critical importance when managing APS patients (1, 2). A most recent investigation over a period of 38 years confirmed that the presence of TP, especially in persistent low-moderate conditions, was strongly associated with poor long-term survival of APS patients (3). However, there is an extreme paucity of effective, safe therapeutic drugs for the long-term management of APS-TP.

Sirolimus, as the mammalian target of rapamycin (mTOR) inhibitor, mainly inhibits cytokine receptor-dependent signal transduction and then blocks the activation of T cells, selectively increasing functional regulatory T cells. In a more recent study, sirolimus has been reported to be effective in connective tissue disease-related TP (CTD-TP) overall, but different types of CTD-TP appear to respond differently to sirolimus therapy (4). So far, there is no relevant study or case report on the efficacy of sirolimus for TP in APS patients, let alone sirolimus monotherapy. Therefore, we launched a pilot project to investigate the efficacy and safety of sirolimus monotherapy for TP in patients with primary APS.

## Materials and Methods

### Study Design and Participants

This is a real-world study in Peking University First Hospital based on our dynamic cohort of APS from January 1, 2020 to December 31, 2021. Inclusion criteria were as follows: (1) with a definite or probable diagnosis (defined as aPL-positive but without classified manifestations) of primary APS, according to the 2006 Sydney criteria for APS (5), (2) aged ≥ 18 years, (3) presented with TP with PLT <100 × 10^9^/l (normal range 125–350 × 10^9^/l), and (4) treated with sirolimus monotherapy for TP. Patients receiving sirolimus clinically for other conditions (e.g., APS nephropathy), or receiving glucocorticoid, immunosuppressive agents concomitantly or in the last 6 months before study entry were excluded. The study was approved by the Institutional Review Board of the Peking University First Hospital. The participants provided their informed consent to participate in this study.

### Clinical Assessments and Data Collection

Baseline data of each eligible participant before initiation of sirolimus were collected, including demographics, symptom duration, APS-related manifestations, laboratory findings, and prior treatment details. After that, all patients were prospectively followed up monthly and then at least 3-monthly when platelet count reached at least 100 × 10^9^/l and their condition was clinically stable. Additional follow-up was scheduled besides those at regular intervals if clinically necessary. The adjusted global APS score (aGAPSS) and damage index APS (DIAPS) were calculated, according to previous literature (6–8). Oral sirolimus was started at a dose of 1–2 mg once daily, and then doses were adjusted primarily based on clinical efficacy and tolerance, with consideration of sirolimus trough concentration of ≤15 ng/ml. The treatment decisions at each visit were made at the discretion of treating rheumatologists in clinical practice.

For both anticardiolipin antibody (aCL) and anti-β2-glycoprotein-I (β2GPI antibody detection, EUROIMMUN^®^ ELISA IgG/IgM test kits were used in a EUROIMMUN^®^ (Lübeck, Germany) fully automatic ELISA Analyzer I. Our local cutoff values for the aCL and anti-β2GPI antibody tests were set as validated manufacturer cutoffs (aCL IgG/IgM >12 GPL/MPL, anti-β2GPI IgG/IgM >20 GPL/MPL). The lupus anticoagulant (LA) test was performed with an ACL Top 700 Werfen^®^ (L‘Hospitalet de Llobregat, Spain) fully automatic coagulometer device with an integrated system of screening and confirmation steps. The APTT-based reagent is used for the antiphospholipid-dependent coagulation technique of the LA test. Confirmation tests were performed in aPTT with hexagonal-phase phospholipids (LA-SCT, Werfen) and in dRVVT with a phospholipid-rich dRVVT reagent (LA-DRVVT, Werfen). Our local cutoff value for the LAC test was set as the >99th percentile of distribution.

### Outcomes and Assessments

In accordance with the immune thrombocytopenia (ITP) International Working Group Criteria (9), complete response was defined as a platelet count ≥100 × 10^9^/l; partial response was defined as platelet count above 30 × 10^9^/l, with at least doubling of the baseline platelet count. Overall response included complete response and partial response. The efficacy measures were the change in platelet count, overall response rate, and complete response rate after sirolimus therapy during the follow-up period. The safety outcomes included tolerance as assessed by the occurrence of common side effects.

### Statistical Analysis

The trends of platelet count changes during the study period were analyzed using generalized estimating equations with an unstructured working correlation matrix and a robust estimation for the covariance matrix. The comparison of platelet count between baseline and month 1 or 3 was performed by using the non-parametric Wilcoxon signed rank-sum test. The cumulative probability of complete response and median time to complete response were calculated according to the Kaplan–Meier method. All the analyses were made using SPSS v.20.0. Microsoft Excel 2010, GraphPad Prism version 8.0, and R software were used to produce the graphs. The level of significance was set at a two-sided p value less than 0.05.

## Results

### Patient Characteristics

In total, 7 consecutive patients with primary APS-TP were included. There were respectively 3 and 4 cases diagnosed as having definite and probable primary APS, according to the 2006 Sydney classification criteria. Of 3 patients with definite primary APS, there were 2 patients with arterial thrombotic events and 1 patient with pregnancy complications. Baseline characteristics of 7 patients before sirolimus therapy are presented in **Table 1**.

**Table 1:** Baseline characteristics of patients with primary APS-TP at enrolment.

Patients	Sex	Age at visit	TP duration (years)	APS types	Comorbidities	Clinical manifestations of APS	Lowest PLT (*10^9^/L)	aGAPSS	DIAPS	aPL profile (ever)	Prior therapies for TP and response	PLT (*10^9^/L) before sirolimus
1	F	61	5	Probable APS	Hypertension	TP	5	17	0	Triple positive	PSL with the highest dose of 30 mg/day (partial response)	59
2	F	58	0.2	Definite OAPS	Hyperthyroidism	Miscarriages, TP	66	4	0	Anti-β2-GPI positive	None	66
3	M	28	0.5	Definite TAPS	None	Stroke, TP, epilepsy	30	9	1	ACL/LA positive	PSL with highest dose of 100 mg/day, RTX, VCR, TPO-RA (partial response)	80
4	M	19	9	Probable APS	None	TP	40	5	0	Anti-β2-GP1/LA-positive	PSL with highest dose of 150 mg/day (no response)	58
5	M	61	1.5	Definite TAPS	Hypertension Hyperlipidemia	Stoke, myocardial infarction, TP	98	17	3	Triple positive	None	98
6	F	58	0.5	Probable APS	Hyperthyroidism depression	TP, Alzheimer’s disease	21	9	0	Triple positive	None	33
7	F	29	1.5	Probable APS	None	TP	50	13	0	Triple positive	None	50

TP, thrombocytopenia; APS, antiphospholipid syndrome; TAPS, thrombotic APS; OAPS, obstetrical APS; anti-β2-GPI, anti-β2-glycoprotein I; ACL, anticardiolipin antibodies; LA, lupus anticoagulant; PSL, prednisolone; MP, methylprednisolone; RTX, rituximab; VCR, vincristine; TPO-RA, thrombopoietin-receptor antagonist.

At the start of sirolimus therapy, the median age of study participants was 58 (range: 19–61) years with a median disease course of 1.5 (range: 0.3–10.0) years. Regarding antiphospholipid antibody profiles, there were 5 out of 7 patients ever with triple positivity during the disease course. At the time of sirolimus therapy, there were 3 patients with triple positivity, 3 with double positivity, and 1 with single positivity. Among the 6 patients with LA positivity, none of them were receiving anticoagulant therapy when LA was tested. The median platelet count was 59 × 10^9^/l (range: 33–98 × 10^9^/l) before sirolimus therapy. Among 7 included patients, 4 patients were treatment-naïve and 3 patients were ever treated for TP, but with partial response in 2 patients and no response in 1 patient. In detail, glucocorticoid monotherapy was applied in 2 patients, and sequential therapies of prednisone, rituximab, vincristine, and thrombopoietin-receptor antagonist were adopted in the remaining 1 patient. The mean (standard deviation) aGAPSS and DIAPS for the included patients were 10.57 (5.29) and 0.57 (1.13), respectively.

In this pilot study, all participants were treated with sirolimus monotherapy for TP and regularly followed up till the final analysis. All the patients completed 6 months of follow-up with a median follow-up of 6 months (range: 6–15 months). In terms of sirolimus regimen, most participants (5/7, 71.4%) were treated with sirolimus at the dosage of 1 mg once daily without dosage adjustment during the entire period of follow-up. For patient 1, the sirolimus dose was escalated from 1 mg once daily at start to 2 mg once daily at 1 month due to insufficient efficacy and tapered to 1 mg once daily at 3 months and 0.5 mg once daily at 15 months due to favorable and stable efficacy, respectively. For patient 3, sirolimus was started from 2 mg once daily, escalated to 3 mg once daily at 2 months due to insufficient efficacy, and tapered to 1.5 mg qd at 7 months due to favorable and stable efficacy (**Table S1**). During the study observation, the trough concentration of sirolimus ranged from 2.6 to 12 ng/ml among the participants. Regarding concomitant therapy for APS, all 7 patients received hydroxychloroquine 200–400 mg/day. Additionally, 2 patients received aspirin and 1 patient received warfarin treatment (**Table S1**).

### Change in Platelet Count

**Figure 1:**
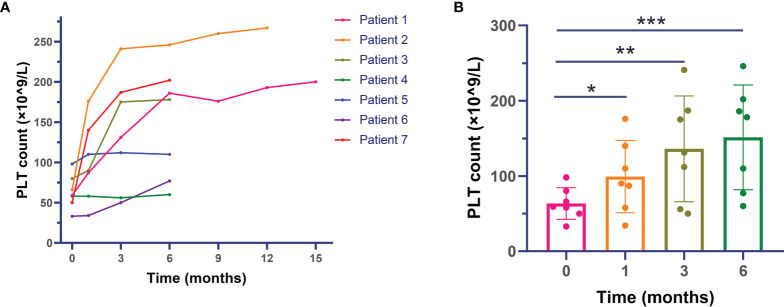
Changes in platelet levels after sirolimus therapy in primary antiphospholipid syndrome patients with thrombocytopenia. **(A)** Platelet count of individual patients during the whole follow-up. **(B)** Median platelet count of included patients during the first 6 months. *: comparison between baseline and month 1 with p < 0.05; **: comparison between baseline and month 3 with p < 0.05; ***: comparison between baseline and month 6 with p < 0.05.

**Figure 1** shows the platelet level before and after sirolimus treatment. Overall, the platelet count of included patients exhibited an increasing trend after administration of sirolimus (p < 0.001). In detail, the platelet count of 59 × 10^9^/l (range: 33–98 × 10^9^/l) before sirolimus therapy was significantly increased to 90 × 10^9^/l (34–176 × 10^9^/l) at the first month (p = 0.028), 131 × 10^9^/l (56–241 × 10^9^/l) at 3 months (p = 0.028), and 178 × 10^9^/l (60–241 × 10^9^/l) at 6 months (p = 0.018) after administration of sirolimus.

**Figure 2:**
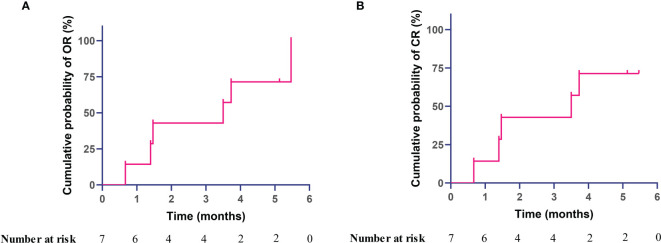
Kaplan–Meier curve with cumulative probability of achieving response after sirolimus therapy in primary antiphospholipid syndrome patients with thrombocytopenia, **(A)** overall response, **(B)** complete response.

Correspondingly, overall response was achieved in 6 out of 7 patients (85.7%) at month 6, with 71% cumulative probability of overall response at month 4 and calculated median time to complete remission of 3.15 months during the treatment observation (**Figure 2**). Complete response was achieved in 5 out of 7 (71.4%) patients. For patient 6 who only achieved partial response but not complete response, the platelet count increased from 33 × 10^9^/l at baseline to 77 × 10^9^/l at month 6. Importantly, overall response was achieved in all of 4 treatment-naïve patients after receiving sirolimus. Of note, all these primary APS patients received sirolimus monotherapy for TP during the entire follow-up period, without any glucocorticoid, immunosuppressants, intravenous immunoglobulins, thrombopoietin-receptor agonist, etc.

### Change in Antiphospholipid Antibody Profiles

**Figure 3:**
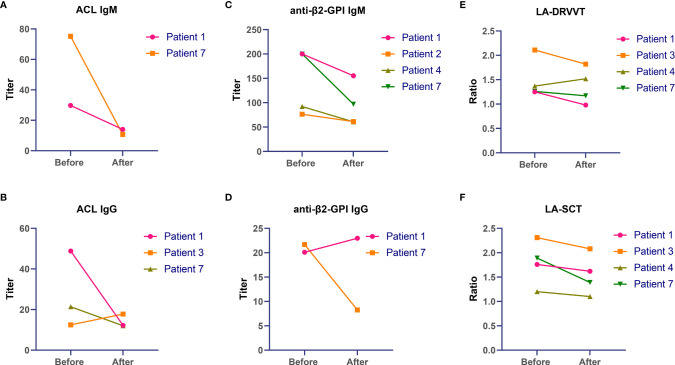
Changes in antiphospholipid antibodies after sirolimus therapy in primary antiphospholipid syndrome patients with thrombocytopenia. **(A)** ACL IgM, **(B)** ACL IgG, **(C)** anti-β2-GPI IgM, **(D)** anti-β2-GPI IgG, **(E)** LA-DRVVT, **(F)** LA-SCT. ACL, anticardiolipin; anti-β2-GPI, anti-β2-glycoprotein I; LA, lupus anticoagulant.

There were 5 patients with repeated testing of aPL antibodies before and after sirolimus therapy. All these 5 patients did not receive anticoagulation therapy. There were different extents of decline in aPL antibodies after sirolimus exposure in all 5 patients as described in **Figure 3** with detailed information provided in **Table S2**. For 2 patients with positive ACL (both IgM and IgG), the titers of ACL antibodies were reduced after sirolimus therapy. For the titers of the anti-β2GPI IgM antibody, declines were observed after sirolimus treatment in all 4 patients with increased level before sirolimus. After sirolimus administration, a decrease in both LA-SCT and LA-DRVVT occurred.

### Safety Measures

The sirolimus regimen was well tolerant. No patients discontinued sirolimus therapy due to safety concerns across the whole follow-up period. There was only a patient experiencing elevated cholesterol level, with low-density lipoprotein cholesterol from 3.14 mmol/l at baseline to 4.15 mmol/l at month 1 and resolve after atorvastatin treatment. No other clinically adverse effect was observed.

## Discussion

In this article, the good efficacy of sirolimus monotherapy for TP is reported for the first time in primary APS patients. After administration of sirolimus, platelet count was raised rapidly in the first 3 months and then increased steadily, yielding an overall response rate of 85.7% and complete response rate of 71.4%, respectively. During the whole follow-up period, no patients discontinued sirolimus therapy due to safety concerns. These results suggest that sirolimus confers good clinical benefits for TP in primary APS with favorable safety and tolerability.

TP occurs in APS with a frequency ranging from 15% to 53%, without a completely clear cause and pathogenesis. TP in APS used to be considered as mild and benign, without the need for additional intervention in most cases. However, accumulating evidence has indicated that the presence of TP was significantly associated with poor long-term survival of APS patients (3). Active management with TP is therefore important to provide a better prognosis for APS patients. In general, glucocorticoid (i.e., prednisone 1–2 mg/kg/day) and high-dose intravenous immunoglobulins are used as the first-line treatment for severe TP in APS (10). However, both are inappropriate for long-term management in clinical practice with high frequency of adverse events and expensive cost. In American Society of Hematology 2019 guidelines for ITP, the task force recommends against a prolonged course of glucocorticoid treatment but is in favor of a short course (≤6 weeks) (11). For those with insufficient response or intolerance to glucocorticoid, immunosuppressants, such as azathioprine and cyclophosphamide, can be considered as second-line medications, but without sufficient supporting evidence for APS patients. Therefore, there is an urgent need to look for effective and well-tolerated treatments for TP in APS patients.

Currently, mTOR pathway activation has been demonstrated as one of critical mechanisms in the pathogenesis of APS (12, 13). Sirolimus, an inhibitor of the mTOR signaling pathway, has been reported for nephrology and cardiac microangiopathy associated with APS in a few studies (14–16). Regarding TP, several lines of investigation have shown that sirolimus treatment is associated with clinical benefits in pediatric ITP and CTD-TP (3, 17). However, the role of sirolimus in TP associated with APS has never been reported so far. In the present study, we found that platelet level was raised rapidly in the first 3 months and then increased steadily, with an overall response rate and complete response rate of 85.7% and 71.4% after sirolimus therapy, respectively. This may be attributed to several possible mechanisms. First, evidence from ITP revealed that impaired autophagy affects the differentiation of hematopoietic stem cells into megakaryocytes and differentiation of megakaryocytes into platelets (18). From this point of view, autophagy target treatment with sirolimus, as the autophagy inducer, has potential to elevate the platelet level in APS-TP *via* promoting platelet release from the bone marrow. Second, robust literatures have demonstrated that ITP is predominately a T cell disorder, characterized by abnormal T-cell responses, particularly defective activity of regulatory T cells. Therefore, novel therapeutics targeting T cells may be the most promising way to cure this disorder (19). Sirolimus mainly inhibits the activation and proliferation of T cells *via* inhibition cytokine receptor-dependent signal transduction but selectively expands functional regulatory T cells (20). Sirolimus hence increases the platelet level by facilitating the development of a regulatory loop of T cells to alleviate the T cell-mediated destruction of platelet. Third, the presence of aPL antibodies has been thought to play important roles in TP associated with APS based on currently available data. The increased expression of platelet membrane glycoproteins and binding of the anti-β2GPI-β2GPI complex after aPL antibody stimulation lead to activation and aggregation of platelets (13). In a previous long-term observational study of 6 consecutive female primary APS patients with severe TP, B-cell depletion therapy with rituximab exhibited greatly sustained clinical efficacy (21). Sirolimus has been confirmed to block B-cell-activating factor-stimulated B-cell proliferation and survival by attenuating mTORC1/2-mediated intracellular free Ca2^+^ elevations and suppressing the Ca2^+^-CaMKII-dependent PTEN/Akt-Erk1/2 signaling pathway (22). Meanwhile, sirolimus is also found to significantly amplify regulatory B cells (23). Clinically, our latest study has comprehensively demonstrated the promising efficacy and good tolerability of sirolimus in patients with systemic lupus erythematosus (SLE) (24). Interestingly, different extents of decline in titers of aPL antibodies were observed after sirolimus exposure in the present study, suggesting that mTOR may play an important role in secretion of antibodies. The presence of aPL antibodies has been confirmed to directly mediate platelet aggregation, and sirolimus may therefore increase the level of platelet *via* inhibiting antibody-mediated causes of TP in APS. Taken together, sirolimus can elevate the platelet count in APS-TP by a variety of diverse mechanisms, including increase in platelet production and reduction in autoimmune-mediated destruction.

Of note, all included patients received the background therapy with hydroxychloroquine, which has been verified to have a multifaceted effect in primary APS, including the thromboprotective role as well as the potential to lowering aPL levels and increasing platelet count (25–27). In a most recent pilot open-label randomized prospective study, long-term hydroxychloroquine exposure was related to a decrease in some of aPL titers over an average 2.6-year follow-up (25). For TP therapy, adding hydroxychloroquine to a second-line treatment for TP has been confirmed as an attractive therapeutic approach in the treatment of children with ITP, particularly in the second line after failure of corticosteroids and/or IVIG (26). In adult patients, the recent study conducted by Khellaf et al. (27) similarly revealed that hydroxychloroquine seems to be a safe and efficacious second-line option for patients with SLE-ITP or ITP and high titer of antinuclear antibodies. At present, the potential to increase platelet count has not been directly confirmed in primary APS patients, which deserves further research in the future.

Experience from organ transplants indicated that the trough concentrations of sirolimus between 6 and 15 ng/ml are appropriate. This has been generalized to other diseases, including ITP and CTD-TP, but with insufficient supportive data. In fact, sirolimus 1–2 mg per day was commonly used for the autoimmune diseases, which is very different from the relatively high-dose regimens of sirolimus (mean daily dose 4.6 mg) in organ transplants (24, 28). In our study, over 80% of the participants attained overall response despite the trough concentrations of 6–15 ng/ml being reached in only 3 patients. This indicates that achieving sirolimus trough concentrations of 6–15 ng/ml may not be generalizable to autoimmune diseases. On the other hand, dose escalation or adjustment primarily based on clinical efficacy and tolerance is appropriate and should be recommended. In the study, sirolimus showed a good safety profile, which was consistent to previous studies (4, 17, 24). The good tolerability of sirolimus observed in our APS patients may be related to the relatively low serum concentration. Monitoring sirolimus trough concentration is still necessary.

There are several limitations that should be acknowledged. First, most of the included patients were with mild or moderate TP, rather than severe TP, because of safety considerations. In renal transplant recipients, the occurrence of TP in the sirolimus, cyclosporine, and prednisone regimen was significantly higher than that of the cyclosporine and prednisone regimen. We therefore selected patients with mild and moderate TP to preliminarily investigate the efficacy and safety of sirolimus for APS-TP in clinical practice. Second, a relative limitation of the study was absence of a control arm for comparison. In fact, to overcome this limitation, we were aided by the APS ACTION Executive Committee and Prof. Chengde Yang who governs the largest APS cohort in China, by providing the predesigned eligible control group treated by glucocorticoid monotherapy or no treatment for TP, although these data were finally failed to analyze due to the low number of eligible participants with necessary data. Other limitations include small sample size. This was just a pilot study, and more high-quality studies are clearly needed in the future to verify our results.

## Conclusion

In conclusion, the pilot study showed good efficacy and tolerance of sirolimus monotherapy for TP in primary APS patients. Application of sirolimus as first-line therapy may be considered. A high-quality study with a larger number of patients is needed in the future to verify our results.

## Data Availability Statement

The raw data supporting the conclusions of this article will be made available by the authors, without undue reservation.

## Ethics Statement

The studies involving human participants were reviewed and approved by the Ethics Committee of Peking University First Hospital. The patients/participants provided their written informed consent to participate in this study.

## Author Contributions

ZZ conceived and coordinated the study, was responsible for the management of some patients, and critically revised the manuscript. WX had full access to all the data collection, analysis, and interpretation and drafted the manuscript. LJ contributed to the study design, follow-up of patients and process of data collection. All authors contributed to the article and approved the submitted version.

## Conflict of Interest

The authors declare that the research was conducted in the absence of any commercial or financial relationships that could be construed as a potential conflict of interest.

## Publisher’s Note

All claims expressed in this article are solely those of the authors and do not necessarily represent those of their affiliated organizations, or those of the publisher, the editors and the reviewers. Any product that may be evaluated in this article, or claim that may be made by its manufacturer, is not guaranteed or endorsed by the publisher.
