# Supplementary material for: Sirolimus Monotherapy for Thrombocytopenia in Primary Antiphospholipid Syndrome: A Pilot Study From a Tertiary Referral Center
Source: Front Immunol. 2022 Mar 25;13:857424. doi: 10.3389/fimmu.2022.857424 (PMC8989728; doi:10.3389/fimmu.2022.857424)
Supplement: Supplementary file 1 [file Table_1.docx]

**Table S1** Sirolimus regimens in the 7 patients with antiphospholipid syndrome

| Patient | Sirolimus regimens | Highest concertation of sirolimus | other concomitant medications |
| --- | --- | --- | --- |
| 1 | Start: 1mg qd;  1 month: 2mg qd (due to insufficient efficacy)  3 months: 1mg qd (due to favorable efficacy)  15 months: 0.5mg qd (due to favorable and stable efficacy) | 7.2 | aspirin + HCQ |
| 2 | 1mg qd, fixed dose | 4.3 | HCQ |
| 3 | Start: 2mg qd;  2 months: 3mg qd (due to insufficient efficacy)  7 months: 1.5mg qd (due to favorable and stable efficacy) | 12 | aspirin + HCQ |
| 4 | 1mg qd, fixed dose | 4.5 | HCQ |
| 5 | 1mg qd, fixed dose | 2.7 | Warfarin +HCQ |
| 6 | 1mg qd, fixed dose | 8.6 | HCQ |
| 7 | 1mg qd, fixed dose | 2.6 | HCQ |

HCQ:  hydroxychloroquine

**Table S2** Changes in titers of antiphospholipid antibodies before and after sirolimus therapy

| aPL | patients | Before sirolimus | After sirolimus | Time interval (months) |
| --- | --- | --- | --- | --- |
| ACL IgM  (<12MPL) | 1 | 29.74 | 14.03 | 15 |
|  | 5 | 29.24 | / | / |
|  | 7 | 75.19 | 10.67 | 3 |
| ACL IgG  (<12GPL) | 1 | 48.81 | 12.33 | 15 |
|  | 3 | 12.47 | 17.77 | 4 |
|  | 7 | 21.35 | 12 | 3 |
| antiβ2-GP1 IgM (<20MPL) | 1 | >200 | 155.24 | 15 |
|  | 2 | 76.38 | 41.52 | 7.5 |
|  | 4 | 92.24 | 60.74 | 6 |
|  | 5 | 56.08 | / | / |
|  | 6 | 81.46 | / | / |
|  | 7 | >200 | 96.91 | 3 |
| antiβ2-GP1 IgG (<20GPL) | 1 | 20.1 | 22.97 | 15 |
|  | 7 | 21.68 | 8.26 | 3 |
| LA-DRVVT  (<1.2) | 1 | 1.25 | 0.98 | 15 |
|  | 3 | 2.11 | 1.82 | 4 |
|  | 4 | 1.37 | 1.52 | 6 |
|  | 5 | 1.79 | / | / |
|  | 7 | 1.26 | 1.17 | 3 |
| LA-SCT  (<1.16) | 1 | 1.76 | 1.62 | 15 |
|  | 3 | 2.31 | 2.08 | 4 |
|  | 4 | 1.20 | 1.10 | 6 |
|  | 6 | 1.42 | / | / |
|  | 7 | 1.89 | 1.39 | 3 |
| anti-PS/PT IgM  (＜30) | 1 | 100.9 | 89.25 | 15 |
|  | 3 | / | 85.7 | 4 |
| anti-PS/PT IgG  (＜30) | 1 | 72.35 | 22.98 | 15 |
|  | 3 | / | 69.12 | 4 |

The antiphospholipid antibody results which are not shown for patients 2, 3, 4, 5 and 6 were negative.

Anti-PS/PT was detected in four patients (patient 1, 3, 4, 7), which was negative in patient 4 and 7.
